# ﻿Population structure of *Taenioides* sp. (Gobiiformes, Gobiidae) reveals their invasion history to inland waters of China based on mitochondrial DNA control region

**DOI:** 10.3897/zookeys.1203.119133

**Published:** 2024-05-30

**Authors:** Chenlian Sun, Zhenming Lü, Jiaqi Fang, Chenhao Yao, Shijie Zhao, Yantao Liu, Li Gong, Bingjian Liu, Liqin Liu, Jing Liu

**Affiliations:** 1 National Engineering Laboratory of Marine Germplasm Resources Exploration and Utilization, College of Marine Sciences and Technology, Zhejiang Ocean University, Zhoushan 316000, China Zhejiang Ocean University Zhoushan China; 2 National Engineering Research Center for Facilitated Marine Aquaculture, Zhejiang Ocean University, Zhoushan 316000, China Zhejiang Ocean University Zhoushan China

**Keywords:** D-loop, eel goby, hydraulic engineering, population differentiation

## Abstract

*Taenioides* sp. is a small temperate fish originally known to inhabit muddy bottoms of brackish waters in coastal areas of China. However, it began to invade multiple inland freshwaters and caused severe damage to Chinese aquatic ecosystems in recent years. To investigate the sources and invasive history of this species, we examined the population structure of 141 individuals collected from seven locations based on partial mitochondrial D-loop regions. The results revealed that the genetic diversity gradually decreased from south to north, with the Yangtze River Estuary and Taihu Lake populations possessing the highest haplotype diversity (Hd), average number of differences (*k*), and nucleotide diversity (π) values, suggesting that they may be the sources of *Taenioides* sp. invasions. Isolation-by-distance analysis revealed a non-significant correlation (*p* = 0.166) between genetic and geographic distances among seven populations, indicating that dispersal mediated through the regional hydraulic projects may have played an essential role in *Taenioides* sp. invasions. The population genetic structure analysis revealed two diverged clades among seven populations, with clade 2 only detected in source populations, suggesting a possible difference in the invasion ability of the two clades. Our results provide insights into how native estuary fish become invasive through hydraulic projects and may provide critical information for the future control of this invasive species.

## ﻿Introduction

Biological invasion is considered one of the leading causes of global biodiversity loss. The successful reproduction and spread of alien species pose a severe threat and lasting impact on the balance of native ecosystems (Roy et al. 2014). Identifying the sources of alien species might help to establish efficient management to prevent invasion (Daane et al. 2018), and determining the invasion routes can improve understanding of potential invasion risks (Corin et al. 2008). Both are vital factors for controlling and managing the spread of invasive species (Hulme 2009; Yue et al. 2021).

The eel goby, *Taenioides* sp., is a newly confirmed candidate species of the genus *Taenioides* (Gobiidae, Amblyopinae) which is frequently mistaken as a form of *Taenioidescirratus* (Yao et al. 2022). It is a small temperate fish initially known to be widely distributed in Chinese coastal waters from the Yangtze River to the Nandu River. It inhabits muddy bottoms in brackish waters areas, such as estuaries, mangrove swamps, and inner bays (Murdy and Randall 2002; Yao et al. 2022). However, massive propagates of *Taenioides* sp. entered major inland freshwaters of China, including rivers, lakes, and reservoirs, making it a common invasive species in recent years. The most affected areas are Taihu, Gaoyou, Luoma, and Nansi lakes and their nearby regions in China (Ni and Wu 2006; Qin et al. 2019). The boom of *Taenioides* sp. is considered a severe threat to local benthic organisms, and it may cause damage to the ecological environment (Liang et al. 2020). Although substantial work on this invasive species’ morphological, behavioral, and physiological characteristics has been described and recorded (Liang et al. 2020; Yao et al. 2022), the sources and mechanisms of the *Taenioides* sp. invasions have never been studied.

The rapid development of molecular biology, coupled with a decreased cost of classical methodologies such as microsatellite, mitochondrial and nuclear DNA sequencing (Darling and Blum 2007; Okada et al. 2007; Chen et al. 2017) has primarily contributed to the extensive use of molecular tools in population genetic structuring analysis. Mitochondrial DNA is applied to be an efficient genetic marker to determine the genetic variation and population structure in native habitats and invasion areas because of their high mutation rate and maternal inheritance (Cameron et al. 2008), which can be further used to infer the source populations (Freshwater et al. 2009; Diaz et al. 2015) and invasion mechanisms of introduced species (Cheng et al. 2008; Betancur-R et al. 2011). The D-loop-containing region is considered the most variable region of mtDNA because of no coding pressure and is widely used to analyze the genetic variation of fish populations (Saeidi et al. 2014; Parmaksiz 2018; Fang et al. 2022). *Taenioides* sp. is an invasive species with short invasion history and presumed tiny genetic difference, of which the D-loop-containing region is sensitive for genetic variation detection.

Here, we assessed the genetic diversity and population structure of *Taenioides* sp. populations collected from the Yangtze River Estuary (YE), to which they are native, and six inland lakes of introduced habitats with mitochondrial D-loop-containing regions. The phylogeographic analysis revealed invasion sources and forces of the *Taenioides* sp. populations in inland freshwaters of China. The results would provide important information for the future control of this invasive species.

## ﻿Materials and methods

### ﻿Sample collection and DNA extraction

**Figure 1.**
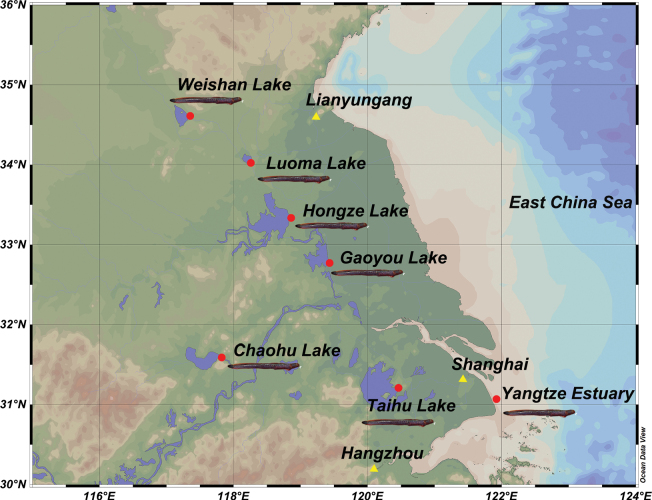
The distribution of sampling locations of *Taenioides* sp.

A total of 141 *Taenioides* sp. samples were collected from seven localities, including Yangtze River Estuary, Taihu Lake (TH), Gaoyou Lake (GY), Hongze Lake (HZ), Luoma Lake (LM), Weishan Lake (WS) and Chaohu lake (CH) during 2021 and 2022, using ground cages (Fig. 1, Table 1). According to our previous study (Yao et al. 2022), all individuals were identified based on morphological and molecular characteristics. Briefly, the total number of vertebrae, dorsal-fin elements, and barbels on the ventral surface of head were counted in morphological observation. Partial fragments of the *COI* gene were amplified and sequenced. Muscle tissues were preserved in 95% ethanol after dissection and transported to the laboratory. The total genomic DNA was isolated following the salt-extraction method (Aljanabi and Martinez 1997). Agarose gel electrophoresis (1.5%) was used to examine the integrity and purity of the extracted DNA. The electrophoresis was performed using 12 μL final volume containing 3 μL extracted DNA, 2 μL 6× loading buffer, and 7 μL double-distilled H_2_O.

**Table 1.** Genetic diversity and neutral test of *Taenioides* sp. populations based on D-loop-containing regions.

Locations	Number of individuals	Code	Number of haplotypes	Haplotype diversity (Hd)	Average number of differences (*k*)	Nucleotide diversity (*π*)	Tajima’s *D*	Fu’s *Fs*
Yangtze River Estuary	29	YE	7	0.7710	5.3000	0.0074	0.5425	3.1047
Taihu Lake	24	TH	6	0.8080	8.7570	0.0122	2.3158	6.5903
Gaoyou Lake	14	GY	4	0.6590	1.0440	0.0015	-0.5601	-0.3268
Hongze Lake	21	HZ	4	0.5330	1.9240	0.0027	-0.0364	1.7497
Luoma Lake	20	LM	6	0.6840	1.8320	0.0025	0.2645	-0.5145
Weishan Lake	20	WS	4	0.4320	1.3680	0.0019	-0.6083	0.7452
Chaohu lake	13	CH	2	0.1540	0.1540	0.0002	-1.1492	-0.5371

### ﻿Mitochondrial DNA amplification and sequencing

Primers (D-loop-F: TTGCCTATGCCATCCTTC; D-loop-R: ATTTGGGCACTTGGTT) were designed using the complete mitochondrial genome sequences of *Taenioides* sp. available from the NCBI (accession number: OL625024) to amplify the target mitochondrial D-loop fragment with Primer Premier (version 6.0) (Premier Biosoft, Palo Alto, CA, USA). The PCR assay was performed in a total volume of 25 μL, which contained 1.25 U Taq DNA polymerase (Promega, USA), 50 ng template DNA, 200 µM forward and reverse primers, 200 µM of each dNTP, 1× reaction buffer and 1.5 mM MgCl_2_. PCR amplifications were performed in a Bio-Rad C1000 Touch Thermal Cycler with the following PCR programs: initial denaturation at 94 °C for 3 min, 34 cycles at 94 °C for 45 s, annealing at 55 °C for 45 s, extending at 72 °C for 90 s and a final extension at 72 °C for 10 min. PCR products were examined by electrophoresis on 1.5% agarose gel and sent to Sangon Biotech (Shanghai) Co., Ltd for sequencing.

### ﻿Data analysis

The obtained nucleotide sequences were aligned using Clustal X (version 1.83) (Thompson et al. 1997) with manual correction. DNA Sequence Polymorphism (DnaSP, version 5.0) (Librado and Rozas 2009) was used to calculate the number of haplotypes (*n*), haplotype diversity (Hd), the average number of pairwise differences (*k*), and nucleotide diversity (*π*) of each population. Phylogenetic analysis was carried out using the maximum-likelihood (ML) method based on the Hasegawa-Kishino-Yano (HKY) model (Hasegawa et al. 1985) with 1,000 bootstrap replicates in MEGA X (Kumar et al. 2018) to determine the genetic relationships of the populations. The Hasegawa-Kishino-Yano model was selected as the best-fit substitution model based on BIC scores among 24 models in MEGA X (Paria et al. 2021). The D-loop control region from *Odontamblyopusrubicundus* and *Amblyotrypauchenarctocephalus*, two species of the same subfamily Amblyopinae, were used as outgroups. The median-joining network of haplotypes was constructed by POPART (version 1.7) (Leigh and Bryant 2015) to estimate the genealogical relationships in *Taenioides* sp. Analysis of molecular variance (AMOVA) was performed using Arlequin (version 3.11) software (Excoffier et al. 2007) to analyze the degree of genetic variability between and within populations. The pairwise genetic differentiation coefficient (*Fst*) values were obtained based on the compute pairwise distances model (Nei and Li 1979) with 10,000 permutations using Arlequin to estimate the genetic differentiation among populations. Pairwise genetic distances between populations were calculated based on the Tamura 3-parameter modeled by using a discrete Gamma distribution (T92+G) (the best-fit substitution model) in MEGA X. IBD (version 1.5.3) (Bohonak 2002) was used for isolation-by-distance (IBD) analysis to evaluate the relationship between geographic and pairwise genetic distances among population pairs. The pairwise geographic distances between each pair of populations were calculated using an online tool (https://www.lddgo.net/convert/distance) based on their latitudes and longitudes. Default parameters were used in Arlequin to investigate Tajima’s *D* test and Fu’s *Fs* test to examine historical population dynamics.

## ﻿Results

### ﻿Population genetic diversity

A D-loop fragment of 722 bp was obtained and analyzed based on 141 sequences from seven populations. Gene sequence analyses revealed that there were 15 haplotypes in the D-loop fragments of the mtDNA (Table 1). A total of 26 polymorphic sites were identified, of which 23 were parsimony-informative sites and three were singleton variable sites. A total of five indel sites were identified. The genetic diversity of seven *Taenioides* sp. populations was analyzed (Table 1). The indices of haplotype diversity (Hd), the average difference (*k*), and nucleotide diversity (*π*) in different populations ranged from 0.1540 to 0.8080, 0.1540 to 8.7570 and 0.0002 to 0.0122, respectively. Among them, the YE and TH populations, two southernmost populations in this study, harbored the highest genetic diversity with the highest Hd, *k*, and *π* values among all locations (0.8080, 8.7570, and 0.0122), and the WS and CH populations harbored the lowest. The genetic diversity of these populations, except for the CH population, showed a decreasing trend from the southernmost population (YE and TH) to the northernmost (WS) from a geographic point of view.

### ﻿Phylogeny and genetic structure

**Figure 2.**
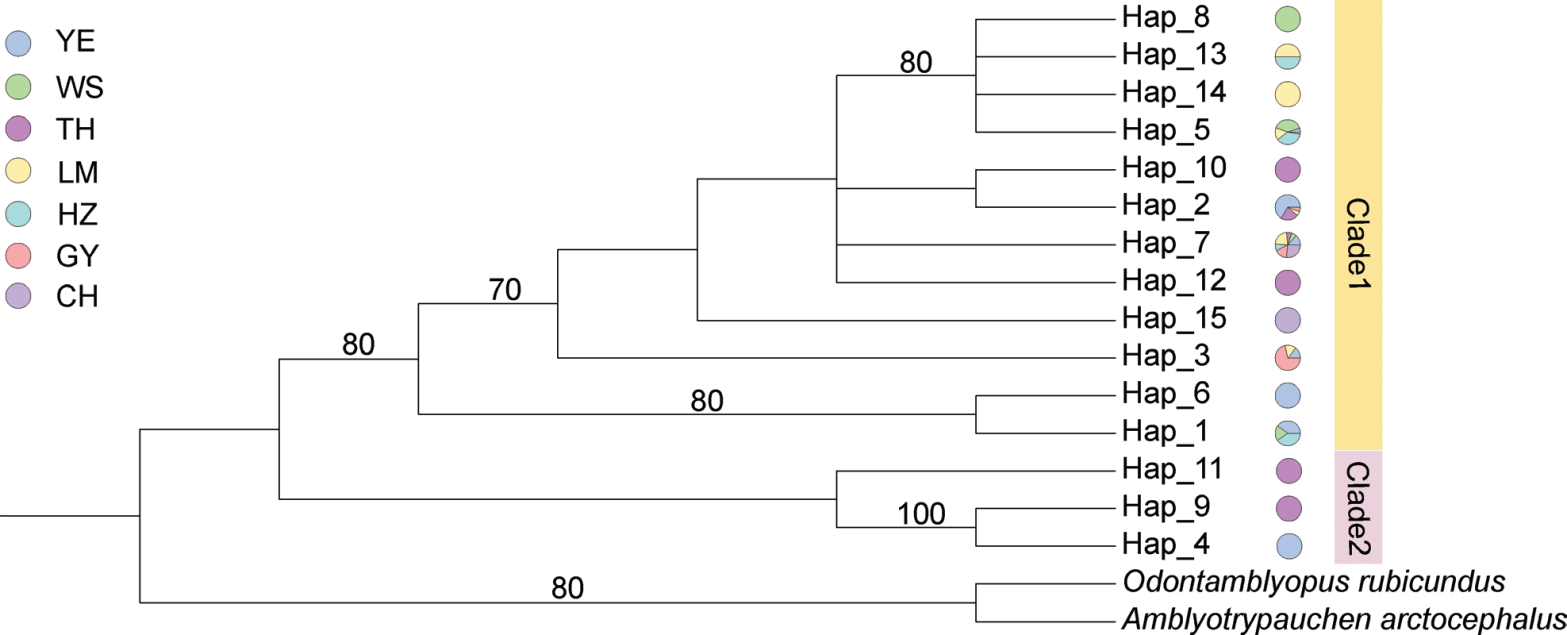
Maximum-likelihood phylogenetic tree constructed from mitochondrial D-loop regions of seven *Taenioides* sp. populations using 15 haplotypes. The number of nodes represents bootstrap values (%) for ML analysis.

According to the sequenced fragments, we built the maximum-likelihood phylogenetic tree with 15 haplotypes in seven populations (Fig. 2). The tree exhibited two main lineages: clade 1 consisted of individuals mainly from the YE, TH, GY, HZ, LM, WS, and CH populations, and clade 2 consisted of individuals only from the YE and TH populations. Hap_5, Hap_8, Hap_13, and Hap_14 formed a subclade in clade 1, consisting of individuals from the YE, TH, HZ, LM, and WS populations. The phylogenetic tree did not separate seven populations.

**Figure 3.**
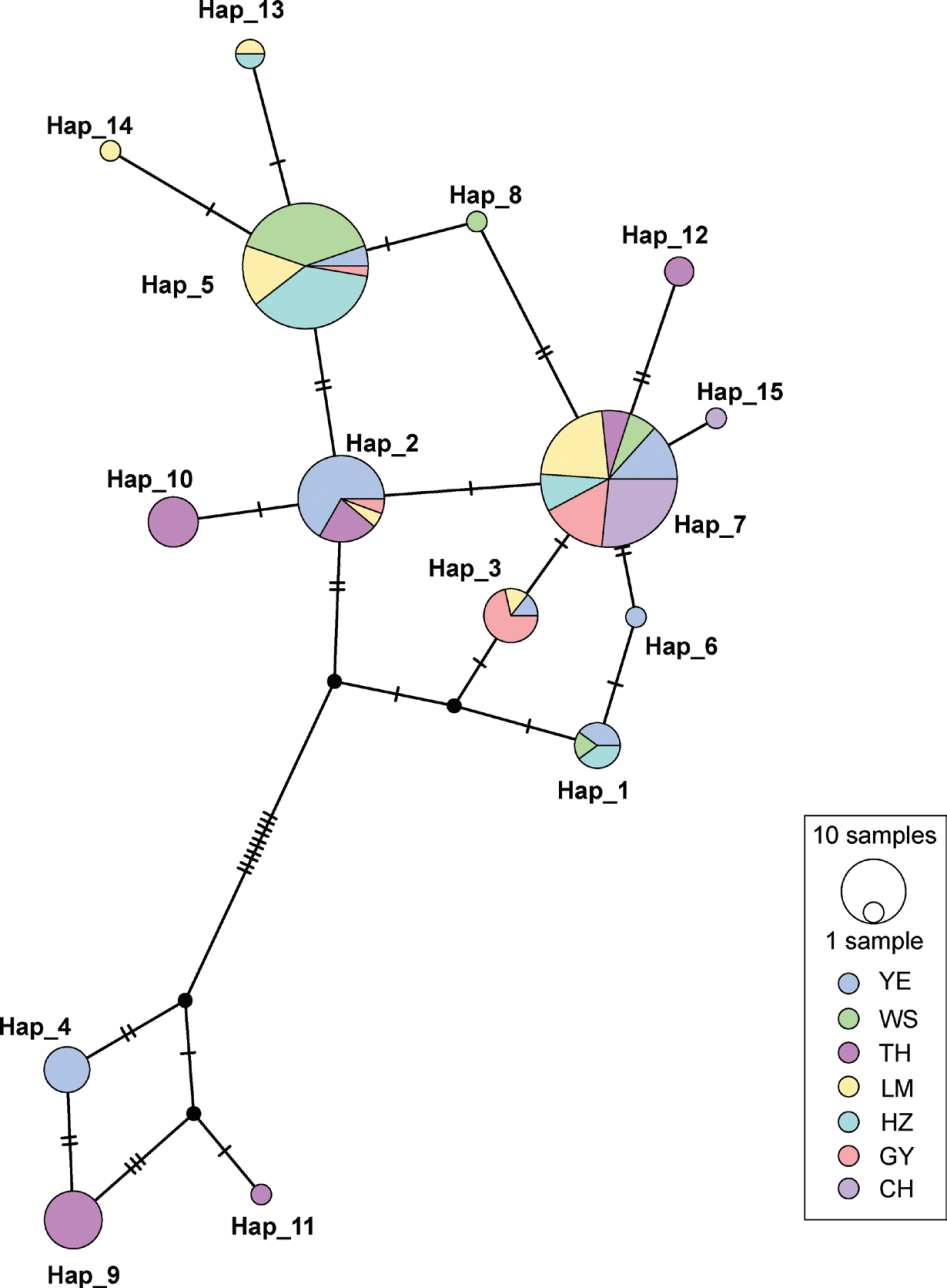
Haplotype network of seven *Taenioides* sp. populations developed with D-loop data. Each circle represents an observed haplotype; the colors reflect the sampling location, the unlabeled small black dots represent missing haplotypes, the small black lines represent the number of mutation steps, and the circle sizes are proportional to the number of samples per haplotype.

The haplotype network could also be divided into two branches (Fig. 3), which showed a consistent topology with two clades of the ML phylogenetic tree. The network showed that the common haplotypes in clade 1 and clade 2 were Hap_7 and Hap_9, respectively. Hap_7 was shared in seven populations in clade 1 and occupied a central position, indicating that it might be the original haplotype. Hap_9 was in only one population (TH) in clade 2. Hap_5 and Hap_2 were also shared by most populations.

The pairwise *Fst* analysis performed on seven *Taenioides* sp. populations showed that *Fst* values ranged from –0.0353 to 0.6670, and the majority of the populations were significantly differentiated (*p* ≤ 0.05) (Table 2). AMOVA analysis based on populations showed significant genetic differentiation (*Fst* = 0.2666, *p* ≤ 0.05) among seven sample locations. The results revealed that 73.34% of the variation occurred within populations, while 26.66% of the variation occurred among populations, suggesting that the genetic variation within the populations was the primary source of total variation (Table 3).

**Table 2.** Pairwise *Fst* (below the diagonal) and genetic distances (above the diagonal) among seven populations of *Taenioides* sp. based on mtDNA D-loop regions.

Populations	YE	TH	GY	HZ	LM	WS	CH
YE		0.0110	0.0053	0.0067	0.0060	0.0068	0.0051
TH	0.0861		0.0105	0.0120	0.0112	0.0120	0.0104
GY	0.1130*	0.2767*		0.0037	0.0025	0.0037	0.0010
HZ	0.2204*	0.3477*	0.4213*		0.0029	0.0022	0.0036
LM	0.1326*	0.3039*	0.1737*	0.0955		0.0027	0.0021
WS	0.2715*	0.3767*	0.5405*	-0.0353	0.1753*		0.0036
CH	0.1674*	0.3085*	0.1610*	0.5414*	0.2805*	0.6670*	

*Statistically significant values (*p* ≤ 0.05).

**Table 3.** AMOVA results among seven *Taenioides* sp. populations using D-loop regions.

Source of variation	df	Sum of squares	Variance components	Percentage of variation (%)	*Fst*
Among populations	6	85.7240	0.6300 Va	26.66	0.2666*
Within populations	134	232.2620	1.7333 Vb	73.34	
Total	140	317.9860	2.3633		

*Statistically significant values (*p* ≤ 0.05).

**Figure 4.**
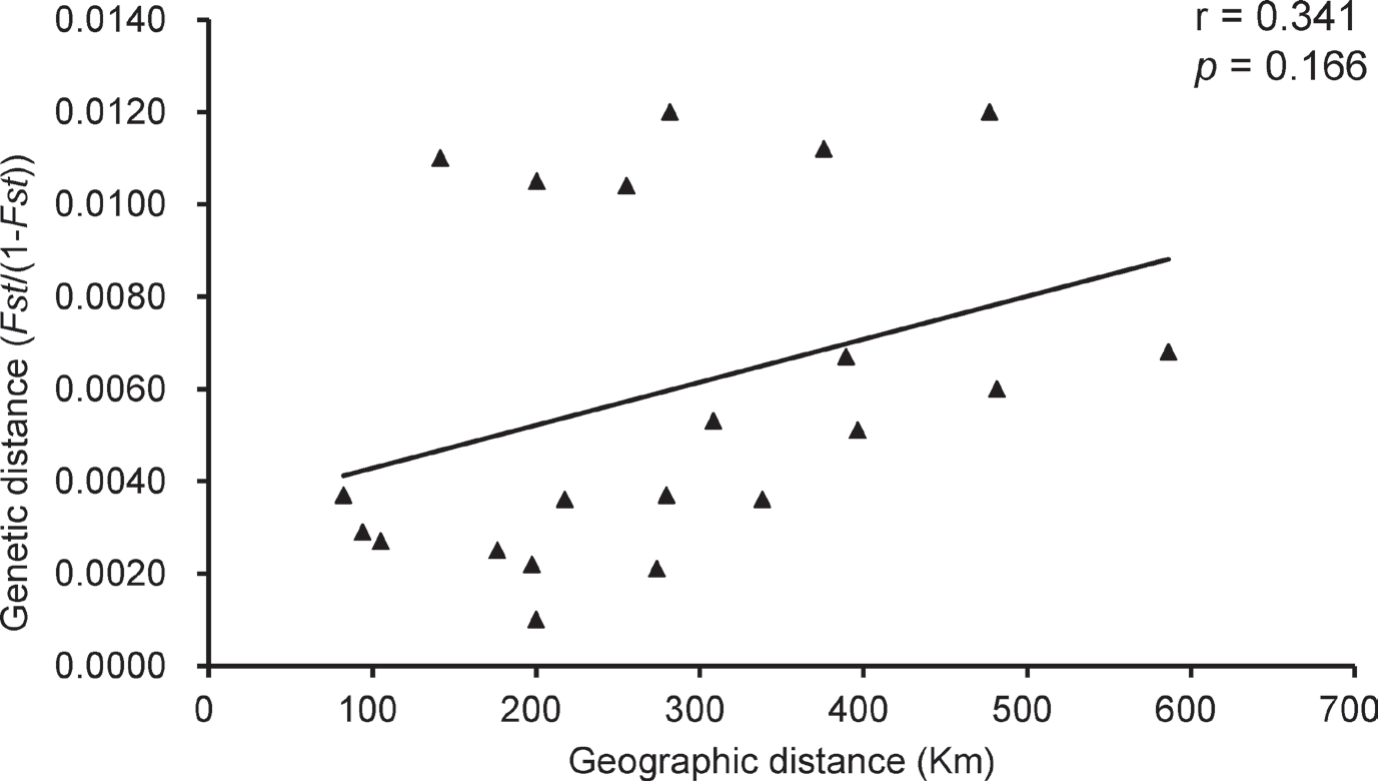
Scatter plots of genetic and geographic distances of *Taenioides* sp. populations.

The sequence analysis results based on the D-loop regions showed that the genetic distances between different *Taenioides* sp. populations ranged from 0.0010 to 0.0120. The IBD results showed moderate (r = 0.341) and non-significant evidence (*p* = 0.166) of a positive relationship between genetic and geographic distances among populations of *Taenioides* sp. obtained from different locations (Fig. 4), revealing that the invasion of *Taenioides* sp. was not driven by active dispersal.

### ﻿Population demographic history

Tajima’s *D* and Fu’s *Fs* neutral tests were used to predict the demographic history of *Taenioides* sp. The results of neutral tests showed that the Tajima’s *D* or Fu’s *Fs* values of the GY, HZ, LM, WS, and CH populations were negative (Table 1), revealing that these populations probably experienced population expansion.

## ﻿Discussion

Genetic diversity generally refers to the sum of the genetic variation of individuals within a species or a population (Wu et al. 2020), which is critical for evolution to adapt to the changing environment. It is traditionally believed that high genetic diversity will help invasive species better adapt to new habitats, making it easier to colonize successfully (Crawford and Whitney 2010). However, during invasions, the genetic diversity of alien species tends to suffer many deprivations, mainly attributed to the founder effects, genetic bottlenecks, or gene drift (Shi et al. 2010). The highest genetic diversity of seven *Taenioides* sp. populations was found in two southernmost sample locations, the Yangtze River Estuary and Taihu Lake. The Hd, *k*, and *π* values of the YE, TH, GY, HZ, LM, and WS*Taenioides* sp. populations showed a descending trend, revealing that the genetic diversity decreased from south to north. This trend is consistent with the regular pattern that the population’s genetic diversity would gradually reduce during the invasion. Combined that the Yangtze River Estuary is one of the conventional habitats of *Taenioides* sp. in China (Wu and Zhong 2008), we speculate that *Taenioides* sp. from the YE and TH populations are the original individuals that invaded the inland waters of China. According to the records, Taihu Lake was first invaded by *Taenioides* sp. in 1960 (Ni and Wu 2006), making it the first inland freshwater lake to be invaded. *Taenioides* sp., which has resided in Taihu Lake for a long period, may have established a relatively stable population capable of maintaining high genetic diversity. *T.cirratus*, one of the representative invasive species of the genus *Taenioides*, has invaded Gaoyou Lake, Luoma Lake, Weishan Lake, and Chaohu Lake (Wang et al. 2017; Qin et al. 2019; Liang et al. 2020), also originating from the Yangtze Estuary and Taihu Lake (Qin et al. 2022). Similar cases have already been described in several invasive species, such as the Europe-introduced red swamp crayfish *Procambarusclarkii* (Barbaresi et al. 2007) and the Japan-introduced bluegill *Lepomismacrochirus* (Yonekura et al. 2007). The CH population, which has the shortest invasion history, may suffer from the most severe founder effect, resulting in the lowest genetic diversity. Considering that the Yangtze River and Taihu Lake are connected by rivers such as the Wangyu River, *Taenioides* sp. in Taihu Lake may come from the Yangtze River Estuary (Dai et al. 2018).

IBD is a common spatial pattern in invasive species (Johansson et al. 2018; Qin et al. 2022). However, IBD analysis for seven *Taenioides* sp. populations detected no significant association (*p* = 0.166) between genetic and geographic distances, revealing that the invasion of *Taenioides* sp. did not follow the active dispersal strategy. IBD is affected mainly by landscape connectivity and invasive species dispersal ability (Sherpa and Després 2021). Combined with that *Taenioides* sp. is a weakly swimming benthic fish (Wu and Zhong 2008), we inferred that active dispersal may not be the only way of invasion. The dam construction of a series of water conservancy projects since the 1960s, such as the water diversion to the northern plains of Jiangsu province, the South-to-North Water Diversion Project, and the water diversion to Chaohu Lake, reconstructed river connectivity and supplied a way for long-distance dispersal of *Taenioides* sp., resulted in a large number of *Taenioides* sp. introductions from the Yangtze River Estuary. Considering that the invasion time of *Taenioides* sp. at each site of the South-to-North Water Diversion Project roughly coincides with the construction time of the project, and the most severely invaded area is also an important node of the project, we infer that human-mediated dispersal likely played an essential role in the invasion of *Taenioides* sp. A similar situation was described in the quagga mussel, a recent invader of the Thames River in Great Britain (Gallardo and Aldridge 2018). There was a severe risk of the mussel further spread through extensive water abstraction of the Thames Valley, which provided a direct link of isolated water basins. Water transfer projects are becoming one of the main approaches to freshwater invasions (Jażdżewski 1980; Leuven et al. 2009; Zhan et al. 2015; Zhao et al. 2019). Therefore, the potential risk of aquatic species invasion should be taken into consideration when constructing water transfer projects.

Interestingly, the *Taenioides* sp. individuals from the source populations (YE and TH) were found to diverge into two branches. One was clade 2, consisting of individuals that did not invade inland waters, and the other was clade 1, consisting of individuals which clustered with those from inland waters, from GY, HZ, LM, WS, and CH populations. Similar situations are common in invasive species, such as *Eurytemoraaffinis* (Winkler et al. 2008). *Eurytemoraaffinis*, a freshwater invasive copepod in the St. Lawrence River drainage basin, exhibited two clades of individuals inhabiting saline-alkali waters in its phylogenetic tree. One of the clades invaded freshwaters with the opening of the waterway, while the other was still limited to its original habitats. The results of physiological studies under a food-insufficient situation showed that some of the individuals from the saline-alkali environment can tolerate fresh water, while others cannot. Hence, we speculated that the reason *Taenioides* sp. from clade 2 failed to invade inland waters is attributed to the enormous differences in salinity between the sea and fresh waters. They failed to adapt to the freshwater environment. More studies are needed to explore the mechanism behind the difference in the invasion ability of the two clades. Individuals from YE, TH, HZ, LM, and WS populations were divided into two branches in clade 1 of the phylogenetic tree (Fig. 2), which suggested a graded invasion of *Taenioides* sp. in inland waters of China. The new subclade consisting of Hap_5, Hap_8, Hap_13, and Hap_14, with a higher degree of invasion, has already formed. To protect the local species diversity, stronger measures should be implemented to prevent the further spread of *Taenioides* sp. in the freshwaters of China.

## ﻿Conclusions

Our study analyzed the genetic diversity and population structure of seven *Taenioides* sp. populations based on mitochondrial D-loop regions. The YE and TH populations held the highest genetic diversity and might be the source of other populations that invaded inland freshwater lakes. Combined with IBD analysis and the history of water conservancy projects, we hypothesized that water diversion may have contributed to the invasion of *Taenioides* sp. in inland lakes. We investigated how water conservancy projects transform an indigenous species into an invasive one and provided insights into assessing the potential impact of water conservancy projects on the natural ecosystem.

## ﻿Additional information

### Author ORCIDs

Chenhao Yao https://orcid.org/0000-0002-1743-2155

### Data availability

Data available in a publicly accessible repository. The data presented in this study are openly available in NCBI at accession numbers PP001065–PP001079.
